# Toxicological Assessment of Cobalt-Chromium Dental Alloys: Ion Release, Cytotoxicity, and Possible Systemic Effects

**DOI:** 10.1007/s12011-025-04938-x

**Published:** 2026-01-08

**Authors:** Ala AM Yahya, Gihan Hosny, Sabah G. El-Banna, Sara A. Alsakhawy

**Affiliations:** Department of Environmental Studies, Institute of Graduate Studies and Research, Alexandria University, Alexandria, 21526 Egypt

**Keywords:** Cobalt-chromium, Cytotoxicity, Artificial saliva, Inflammatory response, Apoptosis, Ion release, Oxidative stress, Hepatotoxicity, nephrotoxicity

## Abstract

Cobalt-chromium (Co-Cr) alloys have broad applications in dental practice because of their mechanical strength and durability. However, the corrosive oral environment promotes ion release, raising concerns about toxicity. This study assessed the cytotoxic, oxidative stress, and inflammatory effects of ions released from Co-Cr alloys. Co-Cr alloy specimens were subjected to a six-month immersion test in artificial saliva, followed by quantification of released ions via Inductively Coupled Plasma Mass Spectrometry. Cytotoxicity was assessed in human fibroblast cells, and systemic toxicity was evaluated in male mice (*n* = 20), divided into control and Co-Cr alloy released ions treated groups. Results revealed a progressive release of Co and Cr ions, peaking at three months. The cytotoxicity assay showed reduced fibroblast viability accompanied by a 2-fold upregulation of the pro-apoptotic marker (Bax) and tumor necrosis factor-alpha, along with a 5-fold increase in interleukin-6 expression. Furthermore, Co-Cr alloy released ions induced a significant increase in oxidative damage, as evidenced by elevated thiobarbituric acid reactive substances and DNA frequency breakage. Antioxidants defenses, including glutathione reductase, catalase, and reduced glutathione, were significantly suppressed in hepatic and renal tissues as compared to the control group. Serum biochemical markers revealed impaired hepatic and renal function, accompanied by dyslipidemia. Histopathological analysis confirmed inflammatory infiltration, hepatocellular degeneration, and tubular alterations. Overall, Co-Cr alloy released ions elicit cytotoxic, oxidative, and inflammatory responses at both cellular and systemic levels. These findings highlight the potential health risks of long-term Co-Cr restorations and emphasize the need for the development of safer alternative biomaterials.

**Supplementary Information:**

The online version contains supplementary material available at 10.1007/s12011-025-04938-x.

## Introduction

Cobalt-Chromium (Co-Cr) alloys are among the most widely used metallic biomaterials in medical fields, such as orthopedics and dentistry, due to their excellent mechanical strength, corrosion resistance, high strength-to-weight ratio, and wear resistance [1, 2]. Co-Cr alloys are critical components in hip and knee joint arthroplasty. However, despite their utility, significant clinical concerns have emerged, primarily related to corrosion at articulating surfaces. The resulting release of fine wear particles and metal ions, particularly Co and Cr, has been shown to cause adverse local tissue reactions and potential systemic toxicity [3]. In dentistry, Co-Cr alloys are a cornerstone material for prosthetics, including removable partial dentures, fixed dental prostheses, and implant frameworks [4]. However, exposure to saliva, dietary components, microorganisms, temperature fluctuations, and pH changes can promote electrochemical degradation, leading to the continuous release of metal ions into the oral cavity. This ion leaching represents a potential biological risk due to its potential to cause local and systemic toxicological responses [5, 6].

Locally, Co and Cr ions have been shown to impair cellular metabolism and proliferation and alter mitochondrial function in oral tissues. Such alterations can lead to reduced fibroblast viability and increased oxidative DNA damage [5]. Meanwhile, Co and Cr ions stimulate reactive oxygen species (ROS) and pro-inflammatory cytokines, a mechanism closely linked to cytotoxicity, genotoxicity, and carcinogenesis [7–9]. Furthermore, Tomova et al. [10] reported that the presence of metal restorations significantly elevates local oxidative stress in the oral cavity, with increased levels of the lipid peroxidation marker 8-isoPGF2-alpha detected in patients’ saliva. Consequently, chronic localized exposure to metal ions may potentiate long-term oxidative damage and oral inflammation, thus predisposing individuals to potentially malignant transformations in the oral mucosa [11]. Beyond these localized effects in the oral cavity, the systemic toxicity of metal ions released from dental alloys has emerged as a critical biomedical concern. Corrosion of Co-Cr prosthetic materials in the oral environment results in continuous ion release, which is then swallowed via saliva, providing a chronic source of systemic exposure [12]. After absorption, Co and Cr ions can circulate systemically and deposit in distant organs, disrupting tissue integrity and impairing organ function [13].

Generally, Co and Cr released ions from implanted alloys exhibited a broad toxicological profile on cardiovascular, neurological, endocrine, renal, and immune systems [14]. Preisser et al. [15] reported the long-term systemic toxicity linked to Co-Cr implants, with elevated systemic metal concentrations lasting years even after implant removal. Furthermore, metal implants can elicit sustained inflammatory responses, driving persistent immune activation that promotes hypersensitivity and bone resorption, ultimately contributing to implant failure [16, 17]. Moreover, Co and Cr ions exhibit strong tissue binding affinity, leading to bioaccumulation in organs such as the liver, kidney, spleen, thyroid, and heart, thus potentially contributing to long-term tissue damage and systemic health risks [18]. In particular, the liver and kidneys, as the primary sites of xenobiotic metabolism and excretion, are particularly vulnerable to oxidative stress, mitochondrial dysfunction and lipid peroxidation [19]. Studies have shown that Co exposure can cause hepatic and renal injury, characterized by impaired organ function, histopathological alterations, and oxidative imbalance [20–23]. Similarly, Cr compounds have been associated with hepatocellular degeneration and nephrotoxicity [24–27].

Despite these well-established systemic risks in orthopedic and biomedical implants, the systemic toxicological impact of ions released specifically from Co-Cr dental alloys remains underexplored. The present work aims to assess the cytotoxic potential of Co-Cr dental alloy released ions in human fibroblast cell lines, along with assessment of the pro-apoptotic protein Bax, inflammatory cytokines, and antioxidant enzymes. In addition, the systemic toxicity of Co-Cr alloy released ions was assessed through functional and histological assessment of the liver and kidneys. Furthermore, the underlying molecular mechanisms were exploited through the measurement of oxidative stress markers, inflammatory mediators, and genotoxic responses following oral administration of Co–Cr ion eluates. By bridging cellular and systemic analyses, this study seeks to provide perspectives into the toxicological profile of dental Co-Cr alloys and highlight potential risks associated with their long-term clinical application.

## Materials and methods

### Materials

A commercially available Co-Cr dental alloy (Nautilus, Bego, Bremen, Germany) was used in this study. The alloy composition consisted primarily of cobalt (63%), chromium (29%), and molybdenum (6.5%), with trace amounts of carbon, silicon, iron, and manganese. Reduced glutathione (GSH), 1-chloro-2,4-dinitrobenzene (CDNB), nicotinamide adenine dinucleotide phosphate (NADPH), and thiobarbituric acid were obtained from Sigma-Aldrich (Saint Louis, USA). Dulbecco’s Modified Eagle’s Medium (DMEM), fetal bovine serum (FBS), and penicillin-streptomycin, were supplied by Gibco (USA). The MTT reagent was obtained from SERVA Electrophoresis GmbH (Germany), while dimethyl sulfoxide (DMSO) and RIPA buffer were procured from Thermo Fisher Scientific (USA).

### Metal Ion Release Profile Assessment

Specimens of Co-Cr dental alloy were prepared for immersion corrosion testing according to ISO 10,271: 2020 [28]. Artificial saliva (pH 4.8) was formulated by dissolving 1.5 g potassium chloride, 1.5 g sodium bicarbonate, 0.5 g sodium dihydrogen phosphate monohydrate, 0.5 g potassium thiocyanate, and 0.9 g lactic acid in a liter of distilled water [29]. Prior to immersion, specimens were cleaned in ethanol for 2 min, rinsed in distilled water, and placed in sterile test tubes. Each sample was placed in a sterile glass tube containing prepared artificial saliva, maintaining a fixed ratio of immersion medium to specimen surface area at 1 mL/cm² to ensure consistent exposure. Triplicate specimens (*n* = 3) were incubated at 37 °C for 3 and 6 months under sterile conditions [30]. Released ions were quantified using inductively coupled plasma mass spectrometry (ICP–MS; Agilent Technologies, USA). Samples were digested with 69% nitric acid (Suprapur, Merck, Germany), diluted, and analyzed after instrument calibration with a multi-element standard containing Co, Cr, Mo, Fe, and Mn (correlation coefficients R² ≥ 0.990).

### Cytotoxicity Assay

Human fibroblasts (ATCC PCS-201-012™) were obtained from the cell culture facility at Center of Excellence for Research in Regenerative Medicine and Applications (CERRMA), Faculty of Medicine, Alexandria University. The cytotoxicity of Co-Cr alloy metal ions was assessed via MTT assay [31]. Cells were maintained in DMEM supplemented with 10% fetal FBS and 0.5% penicillin-streptomycin. Cells were seeded at a density of 7 × 10³ cells per well in 96-well plates and allowed to attach for 24 h under standard culture conditions (37 °C, 5% CO₂). The artificial saliva containing ions released from the Co-Cr alloy after 6 months of incubation at 37 °C was collected, and the concentrations of metal ions were quantified using ICP-MS prior to use in cytotoxicity assays. The solution was then filtered through a 0.22 μm membrane and subsequently diluted in cell culture medium to achieve the desired final concentrations of 10%, 20%, 30%, and 40% (v/v). Blank artificial saliva served as a vehicle control. After treatment, cells were incubated with 100 µL of MTT solution in DMEM for 3–4 h at 37 °C to promote formazan crystal development. The crystals were then dissolved in DMSO, and the absorbance was assessed at 570 nm via an Infinite F15 microplate reader (TECAN, Switzerland). Cytotoxicity assays were performed in triplicate (*n* = 3) for each concentration, and data are presented as mean ± SE.

$$\:\mathrm{C}\mathrm{e}\mathrm{l}\mathrm{l}\:\mathrm{v}\mathrm{i}\mathrm{a}\mathrm{b}\mathrm{i}\mathrm{l}\mathrm{i}\mathrm{t}\mathrm{y}\:\left(\mathrm{\%}\right)\:=\frac{\mathrm{A}\mathrm{b}\mathrm{s}\mathrm{o}\mathrm{r}\mathrm{b}\mathrm{a}\mathrm{n}\mathrm{c}\mathrm{e}\:\mathrm{o}\mathrm{f}\:\mathrm{s}\mathrm{a}\mathrm{m}\mathrm{p}\mathrm{l}\mathrm{e}}{\mathrm{A}\mathrm{b}\mathrm{s}\mathrm{o}\mathrm{r}\mathrm{b}\mathrm{a}\mathrm{n}\mathrm{c}\mathrm{e}\:\mathrm{o}\mathrm{f}\:\mathrm{c}\mathrm{o}\mathrm{n}\mathrm{t}\mathrm{r}\mathrm{o}\mathrm{l}}\:\times\:\:100\:\:\left(1\right)\:$$

### Quantification of Cytokines, Antioxidant enzymes, and Apoptotic Protein

To investigate the molecular response, fibroblast cells were treated for 72 h with 40% (v/v) of artificial saliva containing ions released from the Co-Cr alloy after 6 months of immersion. Following treatment, the culture supernatants were collected and analyzed for interleukin-6 (IL-6) and tumor necrosis factor-α (TNF-α) using Quantikine^®^ ELISA kits (USA) in accordance with the manufacturer’s guidelines. Meanwhile, cell pellets were washed with ice-cold PBS, lysed in RIPA buffer, and centrifuged at 12,000 × g for 20 min at 4 °C. The levels of catalase (CAT), superoxide dismutase (SOD), and Bax protein were determined using commercial ELISA kits (CAT and Bax: Elabscience, China; SOD: RayBiotech, USA). All assays were performed in triplicate (*n* = 3).

### *In vivo* Experimental Design

Adult male mice (30–35 g) were obtained from the Animal House, Faculty of Medicine, Alexandria University. The experimental protocol was approved by the Institutional Animal Care and Use Committee (IACUC) of Alexandria University (Approval No. AU14-250630-1-12) and conducted in accordance with the National Institutes of Health (NIH) guidelines for the care and use of laboratory animals. Male mice were used to minimize hormonal variability, as the estrous cycle in females introduces fluctuations that can significantly alter oxidative stress and inflammatory responses. Mice were maintained at 22 ± 2 °C with free access to water and standard commercial diet (Elfagr Co., El Beheira, Egypt). Each kilogram of feed contained 21% crude protein, 14% crude fiber, and 2% crude fat. Mice were randomly assigned into two experimental groups, with 10 animals in each group. This sample size is common in in vivo studies for evaluating toxicological endpoints, balancing the need for sufficient statistical data with ethical considerations (the 3R principle: Replacement, Reduction, Refinement). Furthermore, with *n* = 10 per group and a standard alpha level of 0.05, the study achieved 80% statistical power to detect a very large effect size (Cohen’s d of approximately 1.3), confirming the adequacy of the sample size for detecting biologically significant toxicological effects [32, 33]. Control group: orally administered 200 µL of artificial saliva. Co-Cr group: orally administered 200 µL of the metal ions released solution from Co-Cr alloy every day for 1 month. The ion-containing solution was obtained from the eluates collected after a 6-month immersion of Co–Cr alloy specimens in artificial saliva at 37 °C. The ion concentrations based on ICP-MS measurements of the immersion test were 0.062 µg/mL Mo, 0.016 µg/mL Fe, 0.0016 µg/mL Mn, and 0.003 µg/mL Cr, equivalent to 12.4 ng, 3.2 ng, 0.32 ng, and 0.6 ng per 200 µL administered dose, respectively.

Following an overnight fast, animals were euthanized via inhalation of an overdose of isoflurane (5%) in a sealed chamber. Euthanasia was confirmed by maintaining isoflurane exposure for one minute following respiratory arrest, immediately followed by a secondary physical method, cutting the major blood vessel aorta in the heart, to ensure irreversible death. Blood was collected from heart, in serum separator tubes to obtain blood serum. Serum was separated by centrifugation at 860 × g for 20 min. Liver and kidney tissues were excised, cleared of adhering fat and connective tissue, and portions of each tissue were homogenized in ice-cold phosphate buffer (0.1 mol/L, pH 7.4). The tissue homogenates were centrifuged at 10,000 × g for 20 min at 4 °C, and the resulting supernatant was preserved at − 80 °C for biochemical assays.

### Assessment of Liver and Kidney Function

Serum biomarkers of hepatic function were determined as follows: aspartate transaminase (AST), alanine transaminase (ALT), and alkaline phosphatase (ALP) were measured using Bio-Diagnostic kits (Egypt), while gamma-glutamyl transferase (GGT) was quantified using a kit from Bio step, Egypt. Renal function was evaluated by determining serum creatinine and uric acid levels with Bio-Diagnostic kits (Egypt), and urea levels with a diagnostic kit from Vitro (Egypt).

### Determination of Lipid Profile

Plasma triglycerides (TG), total cholesterol, high-density lipoprotein cholesterol (HDL-c), and low-density lipoprotein cholesterol (LDL-c) were assessed according to the protocol of Jacklyn et al. [34], Bucolo and David [35], and Assmann et al. [36].

### Determination of Oxidative Stress Biomarkers

Thiobarbituric acid-reactive substances (TBARS), reduced glutathione (GSH) levels were measured according to Tappel and Zalkin [37] and Ellman [38] methods, respectively. Meanwhile, catalase (CAT; EC 1.11.1.6), superoxide dismutase (SOD; EC 1.15.1.1), and glutathione-S-transferase (GST; EC 2.5.1.18) were assayed using the methods of Sinha [39], Nishikimi et al. [40], and Habig et al. [41], respectively.

#### Assessment of DNA Fragmentation

DNA strand breakage was quantified in liver and kidney homogenates using the diphenylamine (DPA) assay [42, 43]. Briefly, 50 mg of tissue was homogenized in Tris-EDTA buffer (pH 8.0) containing 0.2% Triton X-100. DNA fragmentation was assessed by centrifuging the homogenates at 27,000 × g for 20 min, followed by spectrophotometric quantification at 620 nm, and the fragmentation percentage was calculated as follows:

$$\:\mathrm{F}\mathrm{r}\mathrm{a}\mathrm{g}\mathrm{m}\mathrm{e}\mathrm{n}\mathrm{t}\mathrm{e}\mathrm{d}\:\mathrm{D}\mathrm{N}\mathrm{A}\:\left(\mathrm{\%}\right)\:=\frac{\mathrm{T}\:\times\:100}{\mathrm{T}+\mathrm{B}}\:\:\left(2\right)$$

where T denotes the absorbance of the supernatant and *B* denotes the absorbance of the pellet.

#### Measurement of Inflammatory Markers

Interleukin-6 (IL-6) and tumor necrosis factor-alpha (TNF-α) levels were quantified in liver and kidney homogenates using commercially available ELISA kits (CUSABIO^®^, Houston, USA), following the manufacturer’s protocols.

#### Histopathological Analysis

Portions of liver and kidney tissues were excised instantly following sacrifice and fixed in 10% formalin. The fixed samples were dehydrated through a graded ethanol series, and embedded in paraffin wax. Sections of 5 μm thickness were prepared and stained with hematoxylin and eosin (H&E) for histopathological examination [44]. The numbers of inflammatory and pyknotic cells were quantified using ten images obtained from fields for each group under a light microscope. Image analysis was performed using MATLAB software and the ImageJ program. All photomicrographs were captured using 40X objective lens (400 magnification field) with the numerical aperture of a high-resolution (16-bit digital camera, 1280 × 1024 pixels) in the Histochemistry and Cell Biology Department, Medical Research Institute, Alexandria University.

#### Statistical Analysis

All in vitro experiments were performed in triplicate (*n* = 3), and data are presented as mean ± standard error (SE). For the in vivo study, biochemical and oxidative stress parameters were assessed using ten animals per group (*n* = 10). Statistical comparisons between control and treated groups were conducted using an unpaired t-test. A p-value < 0.05 was considered statistically significant. Significance levels in figures are indicated as ** *p* < 0.01 and ***p < 0.001.

## Results and Discussion

### Released Ions from Co-Cr Dental Alloy

**Fig. 1:**
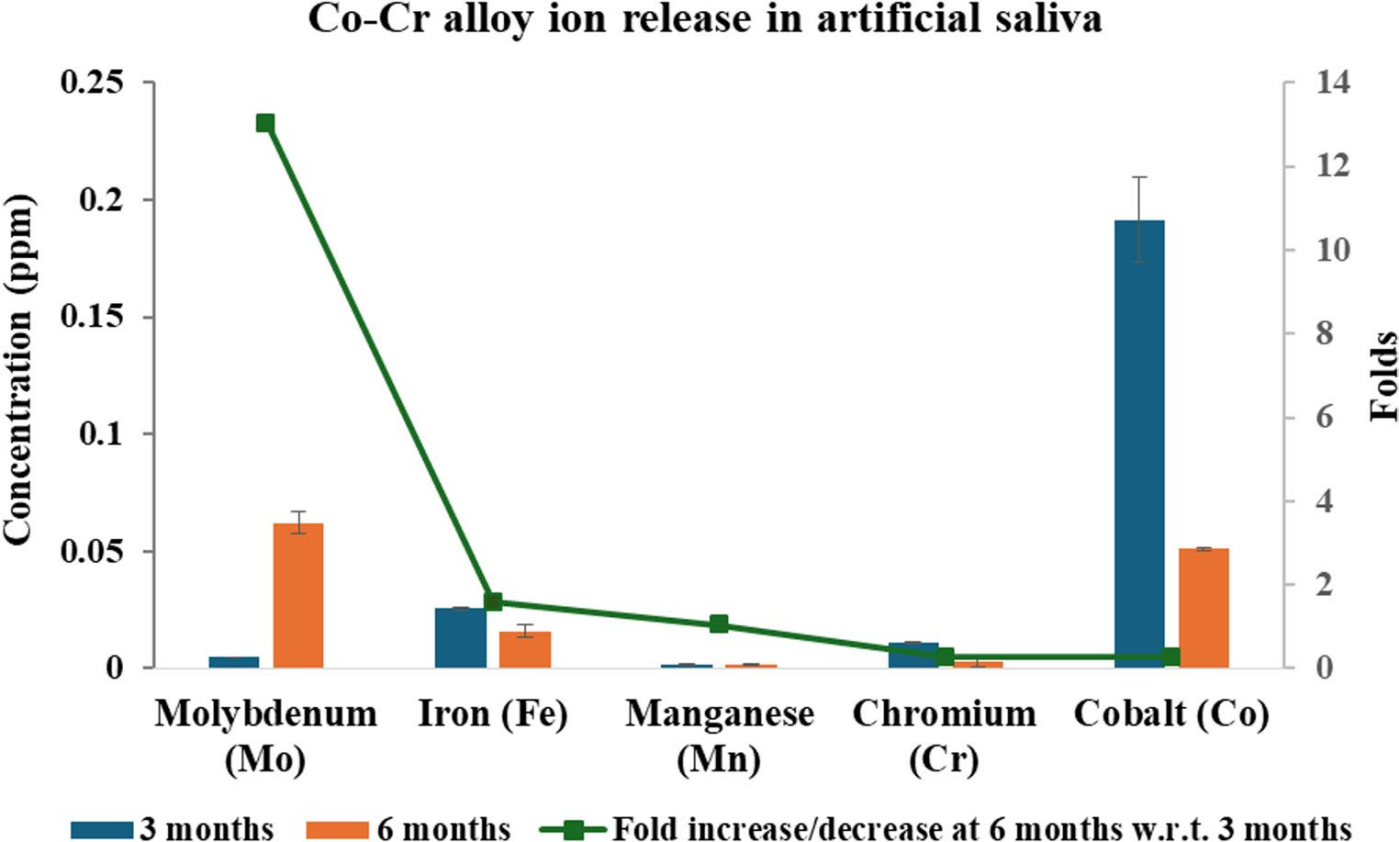
Co-Cr dental alloy released ions after 3 and 6 months of immersion in artificial saliva measured by ICP-MS. Results are expressed as mean ± SE (*n* = 3)

Analysis by ICP–MS demonstrated that immersion of Co-Cr dental alloy resulted in the release of various metal ions, including cobalt (Co), chromium (Cr), iron (Fe), molybdenum (Mo), and manganese (Mn) after 3 and 6 months (Fig. 1). After 3 months, Co showed the highest release (0.191 ppm), followed by iron (0.025 ppm) and Cr (0.011 ppm), whereas Mo and Mn were released at much lower concentrations. Interestingly, after 6 months, the concentration of Co ions markedly decreased by approximately 3.7-fold to 0.051 ppm. In contrast, Mo, which showed a low release at 3 months (0.00476 ppm), increased by about 13-fold at 6 months (0.062 ppm). Iron (Fe) and Cr concentrations also declined at 6 months, with Fe decreasing 1.9-fold and Cr decreasing 1.8-fold. Manganese (Mn) remained nearly stable over time, showing only a minimal change from 0.00155 to 0.0016 ppm.

Generally, Co exhibited the highest release at both time points. The observed fluctuations in ion release over time can be attributed to the dynamic corrosion behavior of the alloy surface. The initial increase in Co levels at 3 months is likely due to early surface corrosion, where loosely bound ions or unstable surface phases dissolve more readily during the first stages of immersion. However, the subsequent decrease in Co, Fe, and Cr concentrations at 6 months may be explained by the gradual formation of a more stable passive oxide layer that limits ion dissolution [45]. In contrast, the marked increase in Mo release at 6 months may be attributed to the selective dissolution or structural rearrangement of the corrosion layer over time.

The present results are in accordance with a previous study reporting similar ion release decrease trends from different orthodontic appliances [46]. In addition, Kassapidou et al. [47] demonstrated that ion release from Co-Cr alloys decreases over time in both physiological and acidic environments, supporting the role of passive film stabilization on the material surface. They also reported that cast Co-Cr alloys exhibited the highest total ion release in acidic conditions, whereas alloys fabricated using advanced techniques, such as laser-sintering, released substantially fewer ions. Furthermore, Sakurai et al. [48] reported that Co-Cr alloys fabricated using additive manufacturing techniques released approximately six-fold fewer metal ions compared to conventionally cast alloys when immersed in acidic saline for 7 days. Accordingly, it can be postulated that surface finishing and manufacturing methods exhibit a critical role in controlling ion release, which complements our findings of time-dependent fluctuations in ion concentrations.

The ingestion of released ions from dental alloys is of considerable concern in terms of long-term biological safety [49]. In this study, ion release was assessed in artificial saliva to simulate the clinical oral environment. However, future research should include alloy submersion in deionized water as a baseline control to better distinguish the inherent dissolution behavior of the material from the effects of corrosive oral conditions. Generally, several factors can influence the corrosion and ion release process, such as elevated chloride content, dietary habits (e.g., intake of acidic foods, fruit juices, and soft drinks), and fluctuations in salivary pH and flow rate [30]. Since the physicochemical properties of saliva vary with diet, health status, and time of day, corrosion behavior in vivo may differ significantly from static laboratory conditions. In the present study, artificial saliva was used under static conditions; however, in the oral environment, higher ion release could be expected due to the continuous flow of saliva, dynamic fluctuations, and the mechanical removal of protective oxide layers during tooth brushing. Previous work demonstrated increased ion release using an oral functioning simulator that more closely mimicked intraoral dynamics [50]. Moreover, surface modifications such as welding or finishing procedures, often performed during clinical use, can induce structural changes including surface roughness, corrosion, and alterations in the crystalline structure, all of which enhance corrosion susceptibility [30].

### Cytotoxicity of Co-Cr Alloy

**Fig. 2:**
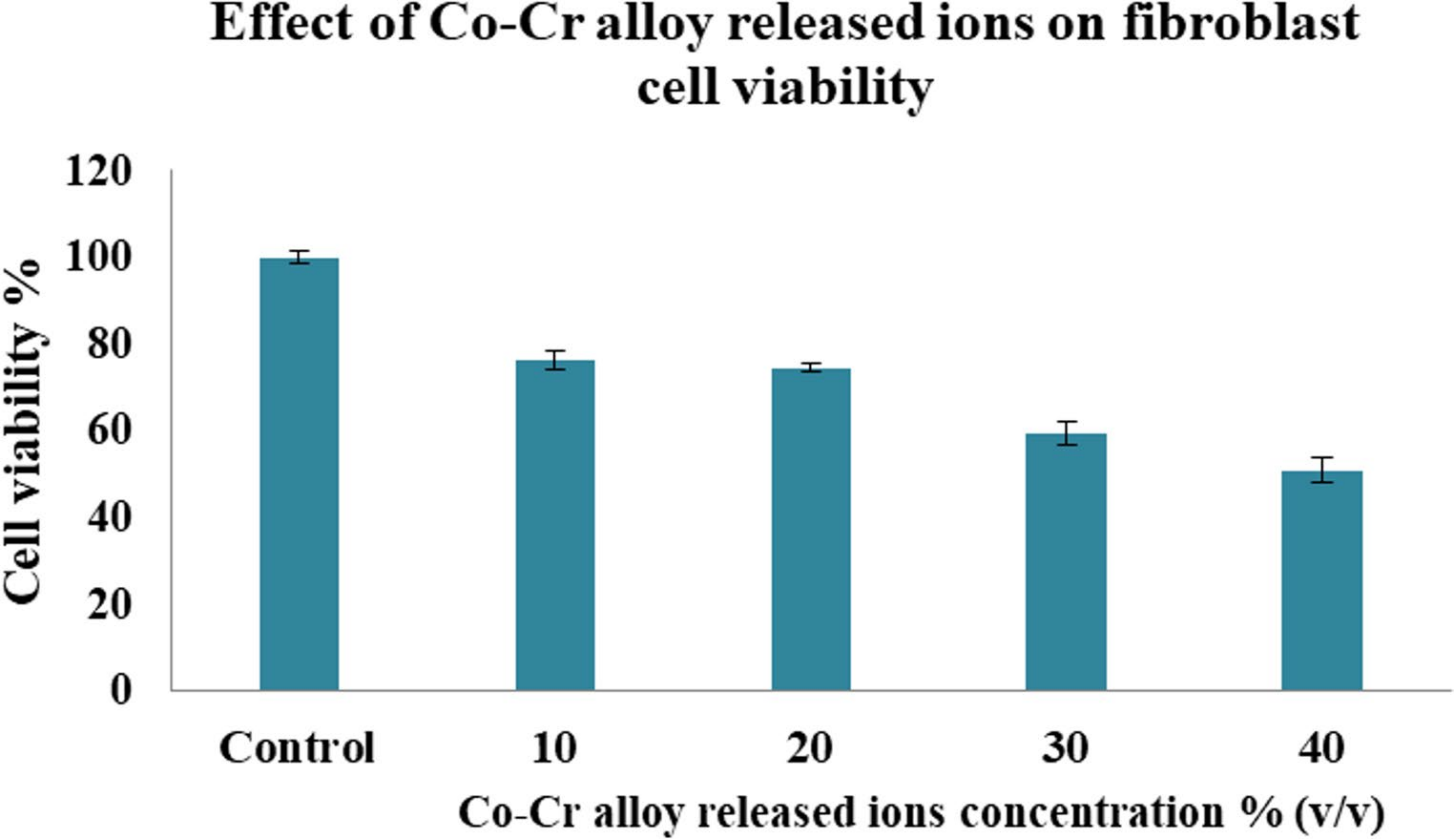
Cell viability of fibroblast cell lines after treatment with artificial saliva containing Co-Cr alloy released ions for 72 h. Results are expressed as mean ± SE (*n* = 3)

The cytotoxicity of Co-Cr dental alloy released ions was assessed utilizing fibroblast cell lines, as they are highly relevant to the biological environment of dental prostheses. Fibroblasts are the predominant cell type in the gingiva, periodontal ligament, and oral mucosa, which are the tissues that have direct and sustained contact with the materials used in removable and fixed dental prostheses [51]. Furthermore, fibroblasts are a standard in vitro model for assessing the biocompatibility and toxicity of dental materials, according to international guidelines for medical device testing (ISO 10993-5) [52] and established practices for dental material biocompatibility assessment (ISO 7405) [9, 53]. The present results revealed that control group treated with artificial saliva exhibited 100% cell viability, indicating optimal cell health in the absence of metal ions. In contrast, higher concentrations of Co-Cr alloy released ions showed progressive reduction in cell viability (Fig. 2). According to ISO 10993-5:2009, cell viability below 70% indicates potential cytotoxicity [52]. In the present study, at concentrations of 30% and 40% (v/v), cell viability percentage was less than 70%, demonstrating a significant cytotoxic effect.

The current findings are consistent with an earlier study that reported Co-Cr alloys trigger cytotoxicity in human gingival fibroblasts (HGFs) under both direct and indirect exposure, with toxicity levels increasing over time [9]. In addition, a previous study reported that Co ions decrease fibroblast viability in a concentration-dependent manner, with prolonged exposure leading to reduced metabolic activity and signs of apoptosis [54]. Similarly, cytotoxicity of Co-Cr alloy has been documented against preosteoblast cells [55] and lymphocytes [56]. Furthermore, McGinley et al. [57] suggested that the cytotoxicity of dental alloys may be exacerbated by local pH reduction, which contributes to unfavorable cellular microenvironments.

### Effects of Co-Cr Alloy on Apoptotic, Oxidative Stress and Inflammatory Responses in Fibroblast Cell Lines

**Fig. 3:**
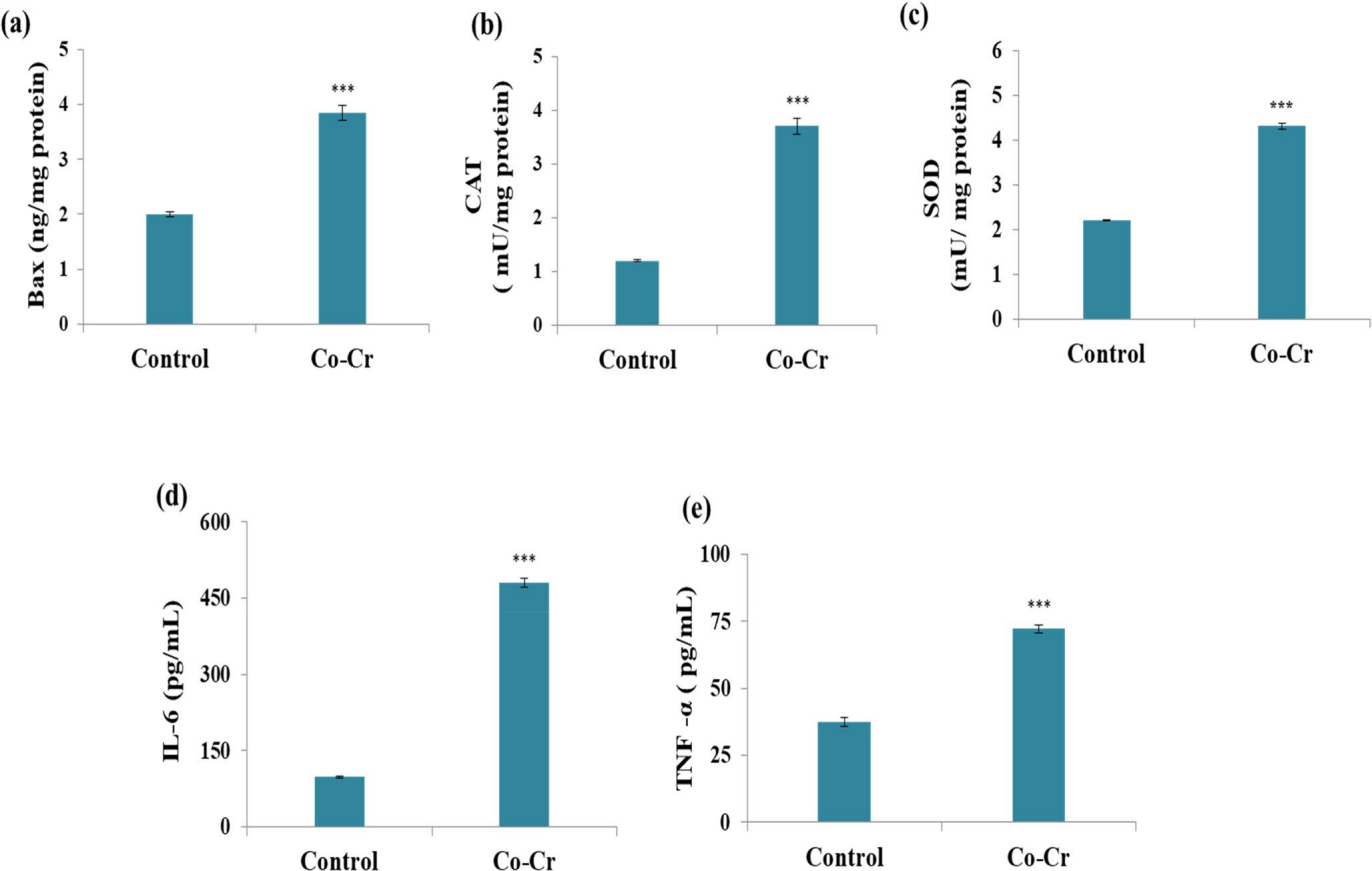
Effects of Co-Cr alloy released ions on (**a**) Bax (**b**) CAT, (**c**) SOD, (**d**) IL-6, and (**e**) TNF-α in fibroblast cell line. Results are expressed as mean ± SE (*n* = 3). ***: significant vs. control at *p* < 0.001, using t-test

To further elucidate the mechanism of cytotoxicity, the pro-apoptotic protein Bax levels were assessed (Fig. 3a). Bax levels were significantly elevated in fibroblasts exposed to Co-Cr alloy released ions compared with the control group. Bax, a central pro-apoptotic regulator within the Bcl-2 family, promotes mitochondrial membrane disruption, thereby activating the intrinsic apoptotic pathway [58]. This finding aligns with the marked decrease in fibroblast viability, suggesting that apoptosis is a major pathway underlying Co-Cr alloy-induced cytotoxicity. The observed upregulation of Bax is consistent with prior reports showing that Co-based materials can trigger mitochondrial-mediated apoptosis. Li et al. [59] reported similar Bax upregulation in rat embryonic cardiomyocytes (H9c2) following cobalt chloride exposure. Liu et al. [60] also demonstrated that Co nanoparticles induced Bax expression in rat liver cells (BRL-3A).

In parallel, antioxidant enzyme activities were examined to assess oxidative stress. Catalase and superoxide dismutase were significantly upregulated in fibroblasts exposed to Co-Cr extracts compared with controls (Fig. 3b&c). CAT and SOD are cellular components responsible for detoxifying hydrogen peroxide and superoxide radicals, respectively [61, 62]. In periodontal tissues, ROS promotes degradation of the extracellular matrix, alveolar bone resorption, and eventual tooth loss [63, 64]. In addition, excessive ROS levels facilitate the release of pro-apoptotic factors and promote apoptosis [65]. These results further support the interrelation of oxidative stress, Bax activation, and cytotoxicity. Similar findings were reported by Li et al. [59], where cobalt chloride exposure upregulated CAT and SOD activities in H9c2 cardiomyocytes.

Inflammatory markers were also evaluated to explore the relationship between metal ion exposure and fibroblast immune responses. Results revealed significant elevation of TNF-α, and IL-6 levels in metal ions treated fibroblasts compared with control cells (Fig. 3d&e). These cytokines are key mediators of inflammation and tissue remodeling. TNF-α is a potent pro-inflammatory cytokine that drives fibroblast activation and contributes to periodontal tissue destruction [66], while IL-6 promotes osteoclast differentiation and bone resorption, thereby accelerating periodontal breakdown [67].

A mechanistic link exists between cytokine production and oxidative stress. Evidence indicates that TNF-α triggers ROS production in epithelial cells [68]. Meanwhile, elevated ROS levels stimulate transcription factors such as NF-κB, and increase TNF-α and IL-6 levels. This establishes a self-perpetuating cycle in which cytokine secretion enhances oxidative stress, and oxidative stress amplifies inflammatory signaling [69]. Together, these processes may exacerbate tissue damage in the peri-implant environment, potentially compromising the long-term stability of dental restorations. Consistent with our findings, Samelko et al. [70] reported synergistic increases in TNF-α and IL-6 when Co particles were combined with bacterial lipopolysaccharides, while Liu et al. [60] demonstrated that Co nanoparticles and Co ions induced marked increases in TNF-α, and IL-6 in liver cells. Overall, the present results demonstrate that metal ions released from Co-Cr alloy exert multi-faceted cytotoxic effects in fibroblasts, characterized by decreased cell viability, upregulation of Bax, activation of antioxidant defenses, and increased secretion of inflammatory cytokines. These interconnected pathways of oxidative stress, apoptosis, and inflammation highlight the potential risks of ion release from dental alloys, as their corrosion and subsequent ion release compromise cellular health and potentially may affect clinical outcomes.

### *In vivo* Toxicity Study

#### Effects of Co-Cr Alloy on Oxidative Stress Biomarkers, DNA Frequency Breakage, and Pro-inflammatory Cytokines

**Fig. 4:**
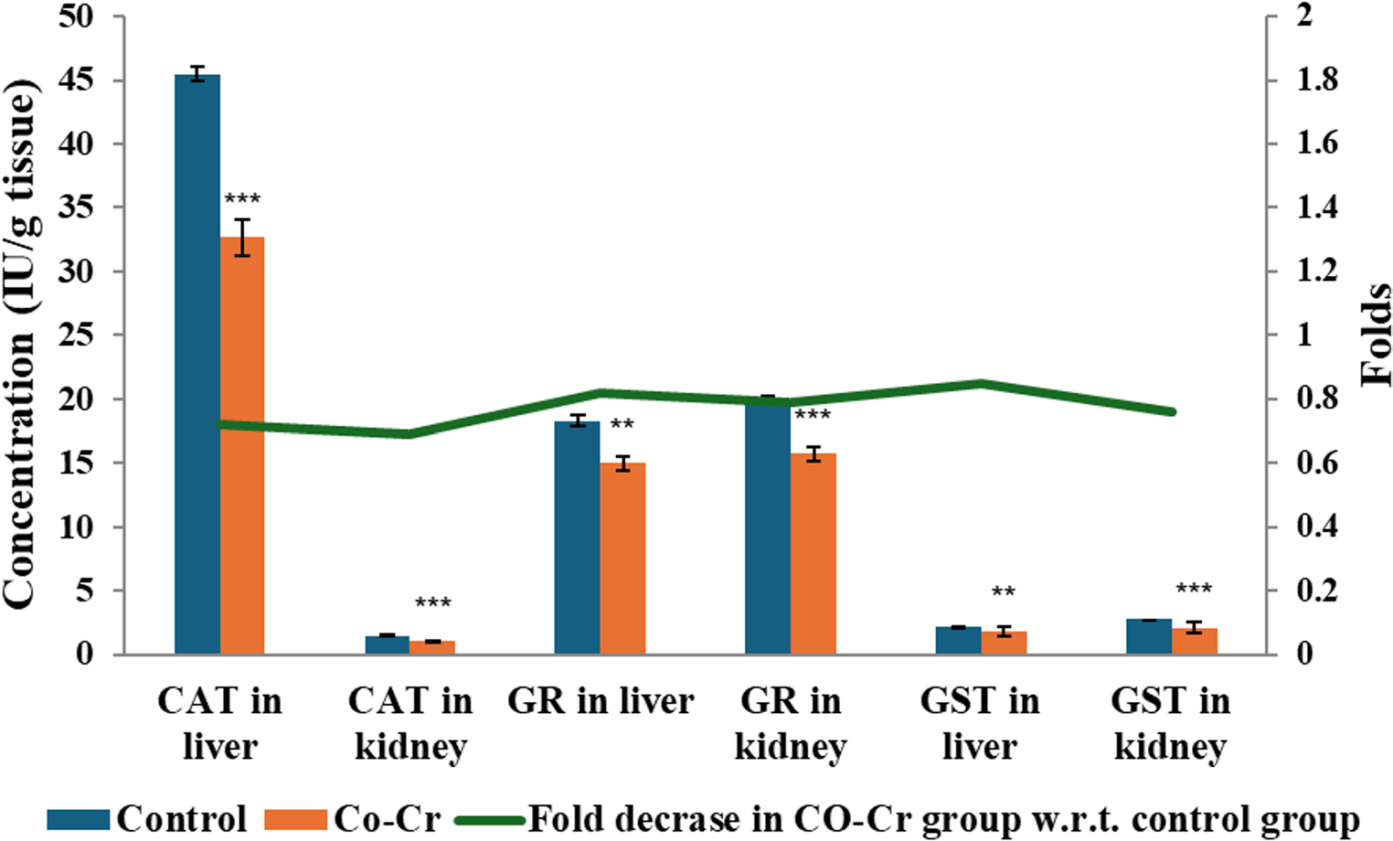
Changes in antioxidant enzymes, including CAT, GR, and GST in liver and kidney of male mice administered Co-Cr alloy released ions. Results are expressed as mean ± SE (*n* = 10 per group). Statistical significance was determined using t-test: **p < 0.01 and ***p < 0.001 compared with the control group

**Fig. 5:**
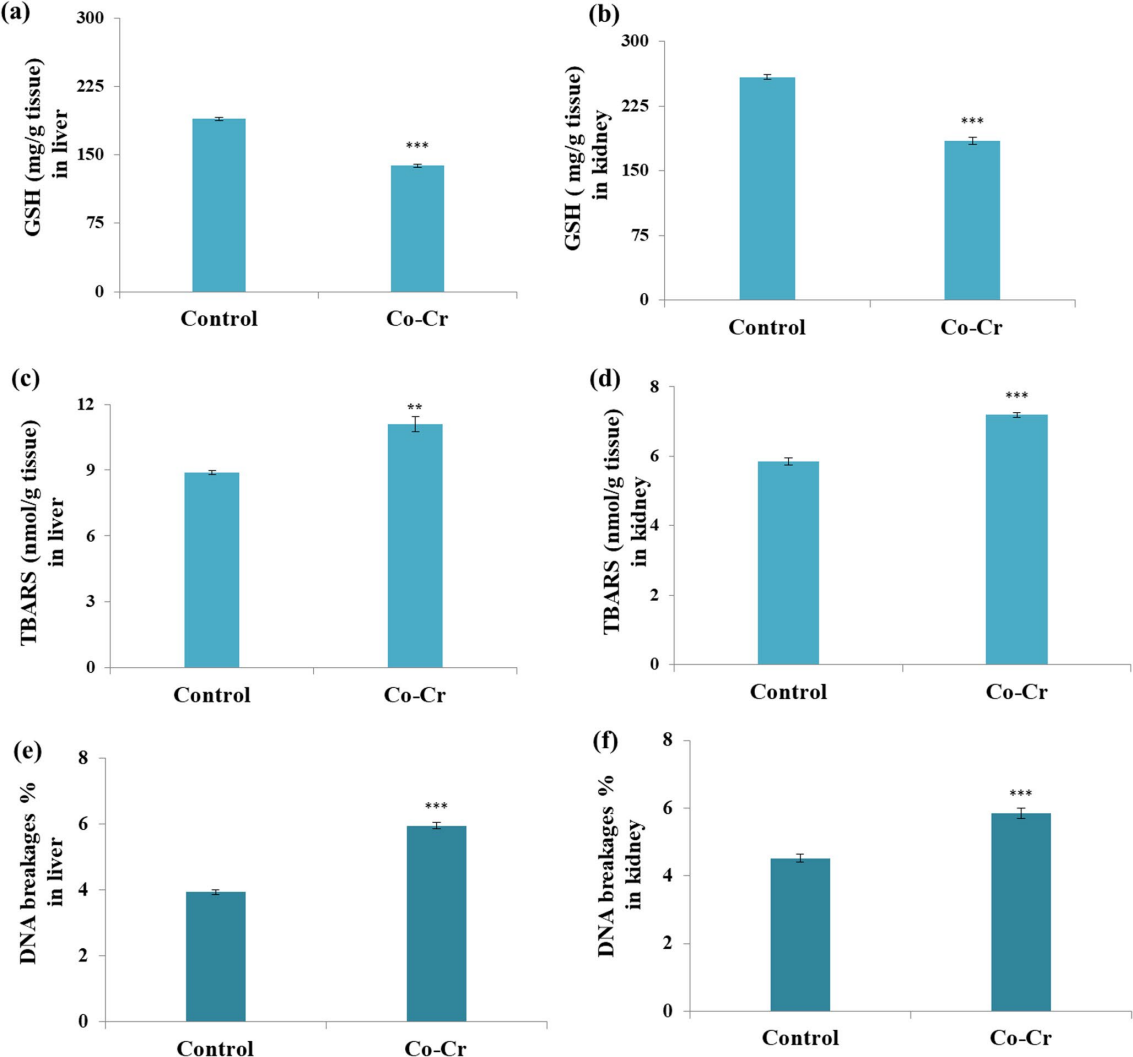
Changes in GSH, TBARS, and DNA breakages % in liver and kidney of male mice administered Co-Cr alloy released ions. Results are expressed as mean ± SE (*n* = 10 per group). Statistical significance was determined using t-test: **p < 0.01 and ***p < 0.001 compared with the control group

Administration of Co-Cr alloy released metal ions significantly disrupted the antioxidant defense system in liver and kidney tissues. As shown in Fig. 4, CAT, GR, and GST activity displayed a significant decrease in the liver and kidney tissues compared with controls. The reduction in hepatic and renal CAT suggests impaired antioxidant defense and heightened oxidative stress susceptibility. Similarly, GSH levels showed a significant decline in the liver and kidney, reflecting depletion of critical intracellular antioxidants responsible for maintaining redox balance (Fig. 5a&b). These results align with previous reports demonstrating Co-induced reductions in antioxidant enzymes in liver and renal tissues [23, 71, 72].

Furthermore, TBARS levels were significantly elevated in Co-Cr group compared with controls, indicating enhanced lipid peroxidation (Fig. 5c&d). Elevated TBARS reflects oxidative degradation of polyunsaturated fatty acids, a key indicator of oxidative tissue damage. This finding corroborates earlier studies in which Co exposure elevated malondialdehyde and hydrogen peroxide in rat kidneys [71] and increased hepatic TBARS in rats exposed to cobalt chloride during gestation and lactation [20]. Bejaoui et al. [73] further reported similar hepatotoxic outcomes upon exposure to cobalt chloride, with elevated malondialdehyde, hydrogen peroxide, protein carbonyls, and decreased antioxidant capacity. Collectively, these findings indicate that Co-Cr alloy released ions exert hepatotoxic and nephrotoxic effects primarily through oxidative stress pathways, depletion of antioxidant defenses, and stimulation of lipid peroxidation.

The effects of Co-Cr alloy released ions on DNA integrity are presented in Fig. 5e&f. The frequency of DNA strand breakages was significantly elevated (*p* < 0.001) in both organs versus the control group, indicating marked genotoxicity. In agreement with these findings, Bijukumar et al. [74] reported that degradation products from Co-Cr-Mo alloys cause substantial DNA damage, including both single- and double-strand breaks, as well as DNA adduct formation in neuronal cells. The genotoxic mechanism is largely attributed to Cr ions, which can induce oxidative stress and subsequently disrupt normal DNA replication and repair processes. Similarly, Co ions have been shown to trigger chromosomal aberrations, DNA strand breakage, and micronucleus formation [75]. These effects are also linked to excessive ROS generation, which directly attacks the DNA backbone and promotes double-strand breaks. Moreover, the strong affinity of Co ions for sulfhydryl groups may impair the function of DNA repair enzymes, further exacerbating genomic instability [75, 76]. Complementing these mechanistic insights, Berniyanti et al. [77] investigated genotoxicity in dental technicians occupationally exposed to metal aerosols and vapors from Co-Cr alloys. Their findings demonstrated significant elevations in key biomarkers of oxidative stress and DNA damage, particularly malondialdehyde and 8-hydroxy-2’-deoxyguanosine in exposed workers. These findings demonstrate the biological impact of metal ion exposure in occupational settings and reinforce the importance of the present study, which models systemic exposure pathways relevant to patients with Co-Cr prostheses. Additionally, Baričević et al. [78] reported genotoxic alterations in the oral mucosa of patients who had worn metal prosthodontic appliances, including Co–Cr–Mo and Ni-Cr alloys, for five years or longer. Accordingly, it can be postulated that exposure to Co and Cr ions, whether through occupational inhalation or through oral and systemic release from dental and orthopedic alloys, activates oxidative stress pathways that trigger DNA fragmentation, impair DNA repair capacity, and increase susceptibility to genotoxic effects in vital organs.

**Fig. 6:**
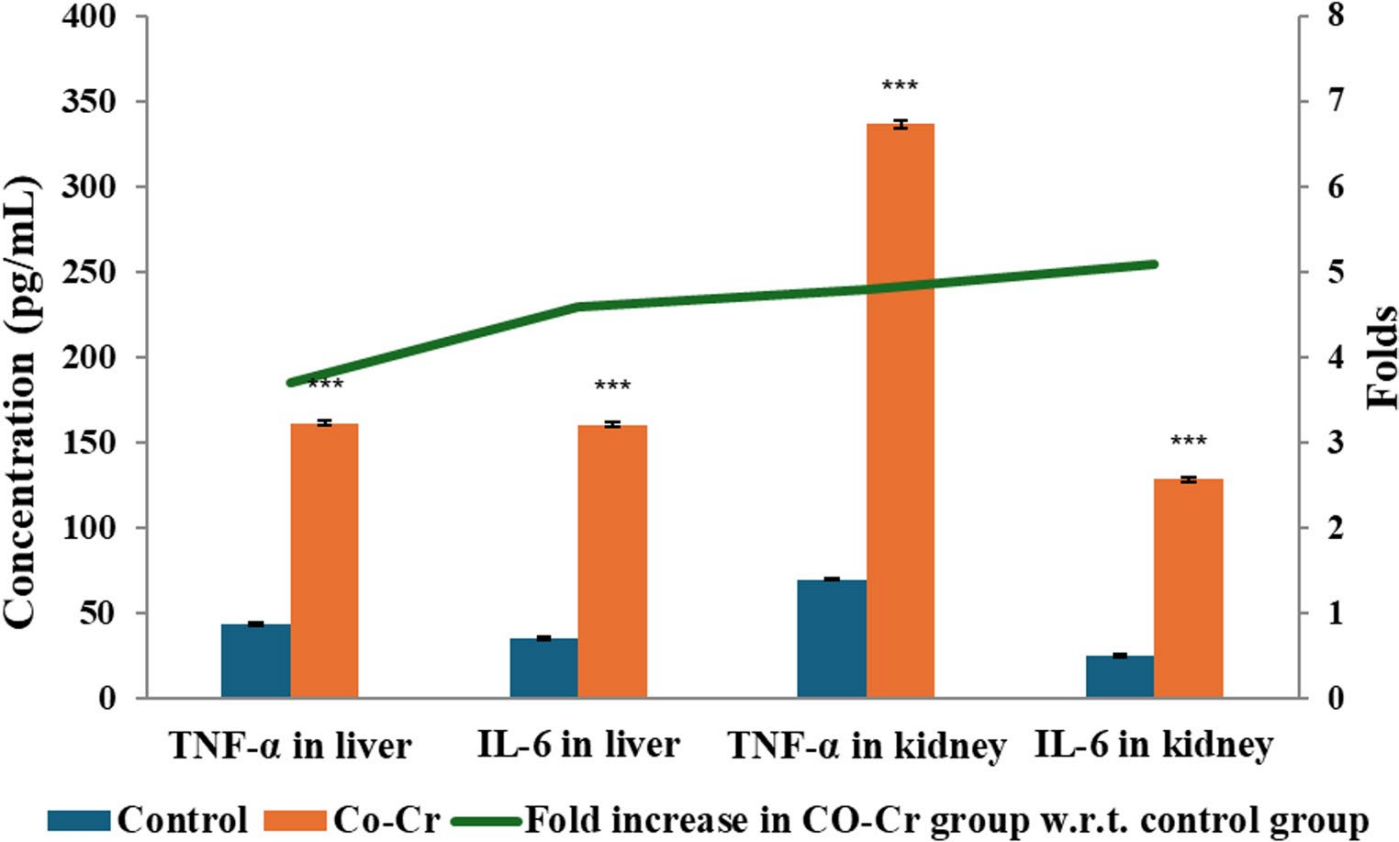
Changes in TNF-α and IL-6 levels in liver and kidney of male mice administered ions released of Co-Cr alloy. Results expressed as means ± SE; *n* = 10 mice each group; ***: significant vs. control at *p* < 0.001, using t-test

Furthermore, TNF-α and IL-6 demonstrated significant elevation (*p* < 0.001) in the Co-Cr group compared with controls, indicating a strong inflammatory response (Fig. 6). These findings are consistent with earlier reports, where cobalt chloride exposure significantly elevated inflammatory mediators [23, 72]. Importantly, TNF-α and IL-6 are upregulated in response to oxidative stress, creating a vicious cycle where inflammation enhances ROS production, and ROS in turn amplify inflammatory signaling [79]. The convergence of oxidative stress, lipid peroxidation, DNA damage, and cytokine overproduction strongly indicates that the hepatotoxic and nephrotoxic effects of Co-Cr alloy ions are mediated through interlinked oxidative and inflammatory pathways. Consistently, Liu and Guo [80] reported that during periodontitis, excessive ROS production overwhelms the natural antioxidant defenses, leading to redox homeostasis disruption, lipid peroxidation, protein oxidation, and DNA damage. Furthermore, this oxidative imbalance activates NF-κB, promoting the release of pro-inflammatory cytokines such as IL-6 and TNF-α, thereby intensifying inflammatory responses and tissue degradation.

#### Effects of Co-Cr Alloy on Liver and Kidney Function Parameters

**Fig. 7:**
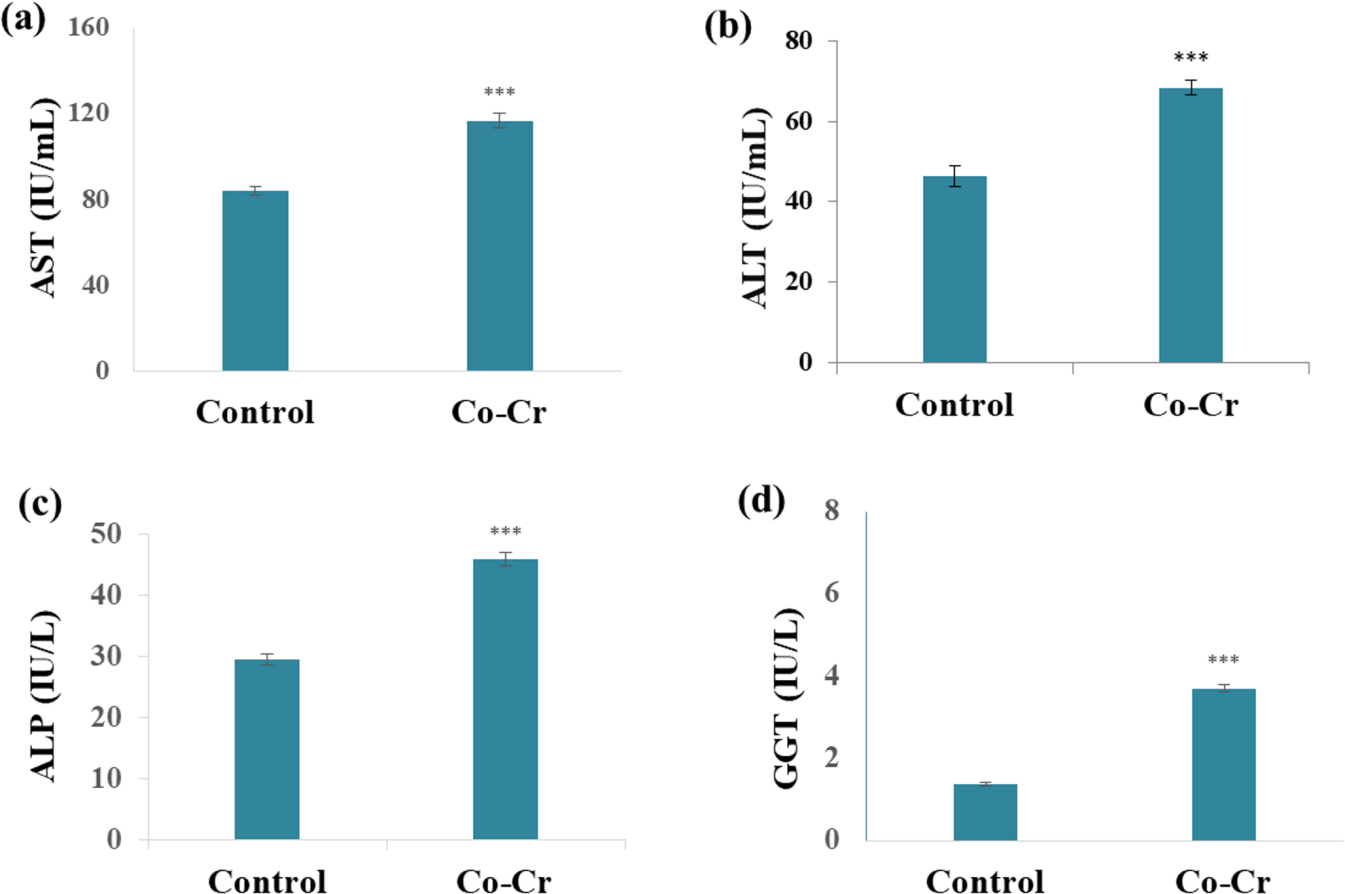
Effects of Co-Cr alloy released ions on (**a**) AST, (**b**) ALT, (**c**) ALP, and (**d**) GGT in blood serum of male mice. Results are expressed as mean ± SE (*n* = 10 per group). ***: significant vs. control at *p* < 0.001, using t-test

The results revealed a significant elevation (*p* < 0.001) of all biochemical markers, including AST, ALT, ALP, and GGT in the Co-Cr group, suggesting hepatotoxicity and systemic biochemical disturbances (Fig. 7). ALT, and AST, found both in the cytoplasm and primarily in the mitochondria, play essential roles in catabolic processing of amino acids and the formation of glutamate and pyruvate for ATP synthesis [81]. Under normal conditions, these enzymes remain confined within hepatocytes; however, hepatic injury disrupts membrane integrity, resulting in leakage of ALT and AST into the circulation and elevated serum levels [82]. Our findings are consistent with Isik et al. [23] study reporting that Co exposure in animal models caused significant increases in ALT and AST activities, indicating direct hepatocellular damage. Similarly, Iji et al. [21] reported that rats treated with cobalt chloride exhibited marked elevations in ALT, AST, and ALP, further supporting Co-induced liver injury. Furthermore, the elevation of ALP and GGT in the present study also reflects potential biliary dysfunction, as these enzymes are strongly linked to bile duct injury and oxidative stress-related hepatobiliary impairment [81].

**Fig. 8:**
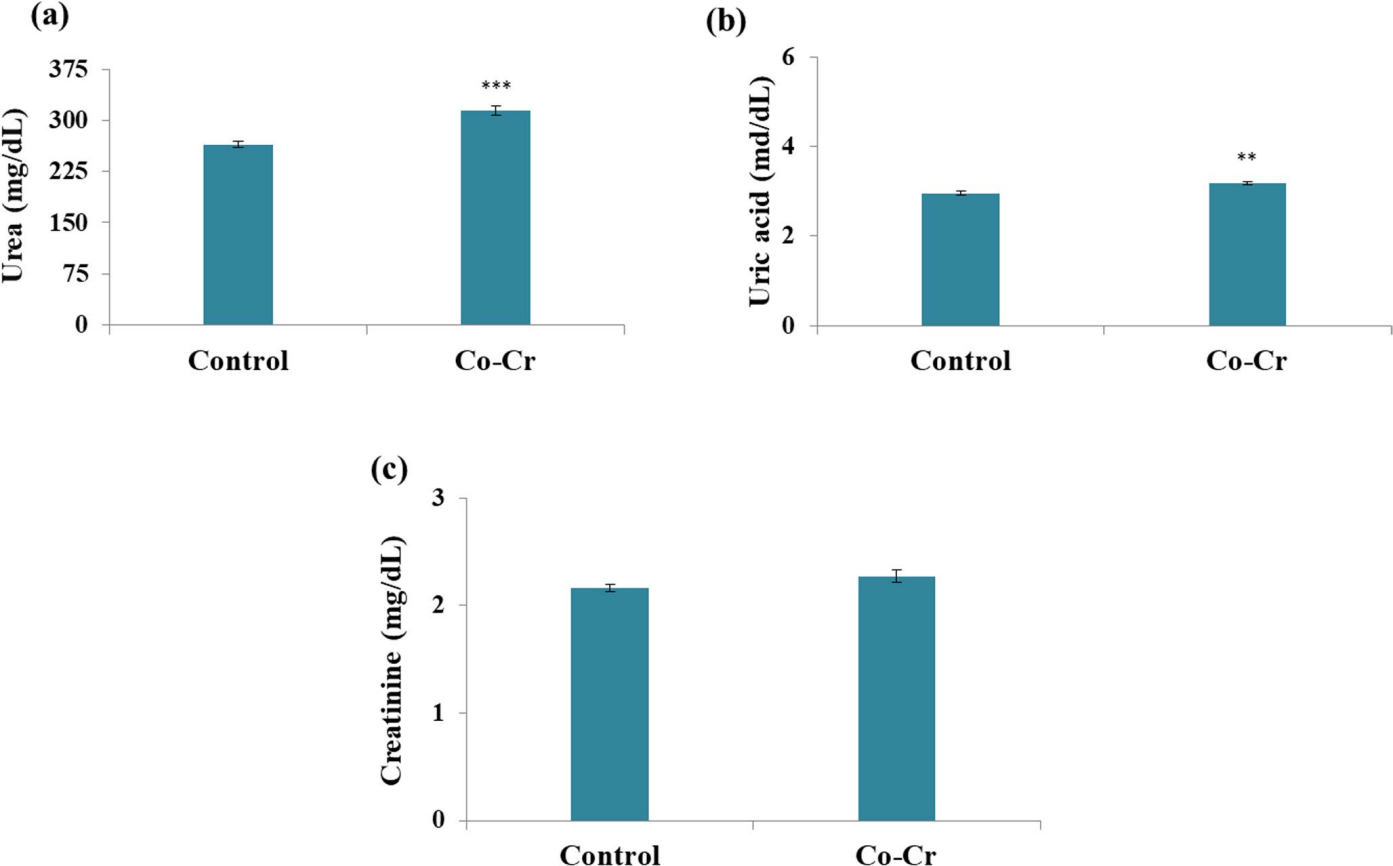
Effects of Co-Cr alloy released ions on (**a**) urea, (**b**) uric acid, and (**c**) creatinine in blood serum of male mice. Results are expressed as mean ± SE (*n* = 10 per group). Statistical significance was determined using t-test: **p < 0.01 and ***p < 0.001 compared with the control group

The present findings revealed a significant increase in urea, and uric acid levels in the Co-Cr group, while creatinine levels insignificantly changed compared to the untreated mice (Fig. 8). Urea and uric acid are important markers of renal function, where their elevation in the Co-Cr group indicates impaired renal clearance. Interestingly, serum creatinine levels remained statistically unchanged, which may imply that glomerular filtration was not severely affected or that tubular dysfunction preceded glomerular damage [83]. The current findings are consistent with Ajibade et al. [84] study, which reported an increase in urea levels upon cobalt chloride treatment for two weeks. Furthermore, Iji et al. [21] found a significant elevation in both bilirubin and creatinine in rats exposed to 300 ppm cobalt chloride daily for seven days, suggesting that higher doses may cause more pronounced glomerular damage compared to our findings.

#### Effects of Co-Cr Alloy on Lipid Profile

**Fig. 9:**
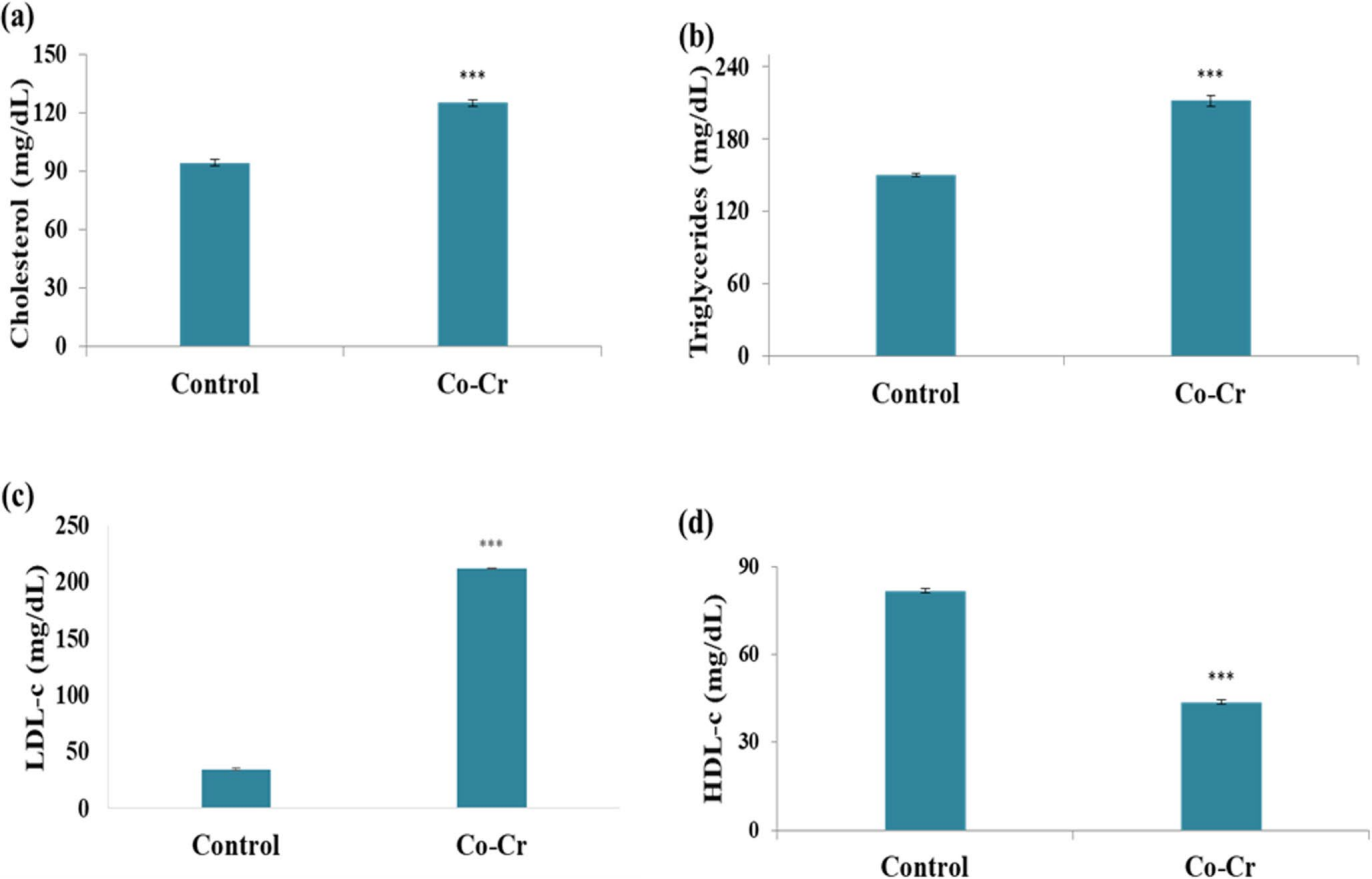
Effects of Co-Cr alloy released ions on (**a**) cholesterol, (**b**) triglycerides, (c) LDL-c, and (d) HDL-c in blood serum of male mice. Results are expressed as mean ± SE (*n* = 10). ***: significant vs. control at *p* < 0.001, using t-test

The results demonstrated a significant elevation (*p* < 0.001) in serum levels of total cholesterol, triglycerides, and LDL-c in the Co-Cr group when compared with controls (Fig. 9). In contrast, HDL-c levels were significantly decreased. Similarly, Wang et al. [85] observed increased total cholesterol and LDL-c, while decreased HDL-c levels upon increasing Co concentrations. These alterations may be attributed to Co-induced oxidative stress, which results in lipid metabolism impairment [23, 71]. Furthermore, Vladov et al. [86] reported that cobalt exposure disrupts systemic metabolism leading to dyslipidemia and altering blood biochemical parameters. Their results align with the elevated cholesterol and LDL-c levels observed in our study, inferring that absorbed Co ions can impair lipid regulation and promote metabolic imbalance.

#### Effects of Co-Cr Alloy on Liver Histopathology

**Fig. 10:**
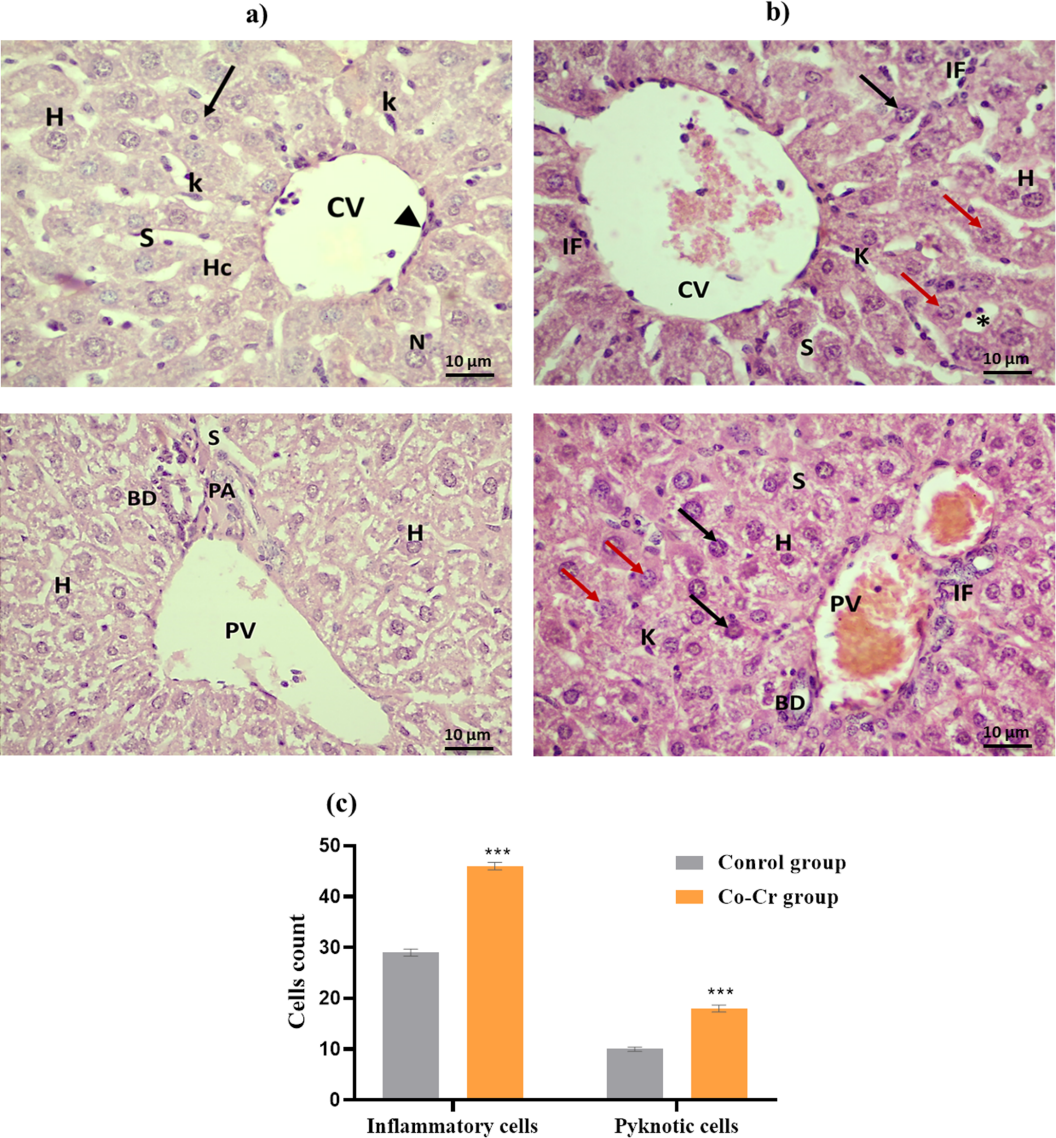
Photomicrographs of liver from (**a**) Control group, (**b**) Co-Cr alloy ion released treated group stained with H&E, and (**c**) Quantification of inflammatory and pyknotic cells in hepatic tissue across experimental groups. Bars represent the mean ± SE (*n* = 10). ***: significant vs. control at *p* < 0.001, using t-test. Control group shows strands of hepatic cords (HC) composed of hepatocytes (H) with their nuclei (N) and binuclear hepatocyte (arrow). The central vein (CV) is outlined by a thin endothelium consisting of simple squamous epithelial cells containing flattened nuclei (head arrow). The portal tract is composed of portal vein (PV), portal artery (PA) and bile duct (BD); Notice, blood sinusoid (S) and Kupffer cells (K) are also observed. Co-Cr alloy treated group shows mild inflammatory cells infiltration (IF) around hepatic tissue and portal tract (PT) with congested portal vein (PV) and obstruction of bile ducts (BD); Some hepatocytes (H) with vacuolated cytoplasm and pyknosis (black arrow), or karyolysis (red arrow) (Scale bar 10 μm, magnification x400)

The control group exhibited typical liver structure, with hepatocytes containing centrally located round nuclei with prominent nucleoli and homogeneous cytoplasm, reflecting normal cellular function (Fig. 10a). The central veins, portal triads, and sinusoids appeared intact, with no evidence of congestion, dilation, or hemorrhage. Kupffer cells were observed along the sinusoidal walls in normal numbers and morphology, while bile ducts maintained uniform epithelial linings without pathological alterations. Importantly, no fibrosis, necrosis, fatty degeneration, or inflammatory cell infiltration was detected, confirming preserved hepatic structure and function in the untreated animals.

In contrast, liver tissues from Co-Cr ion treated mice demonstrated significant histopathological alterations indicative of hepatotoxicity (Fig. 10b). The hepatic cords appeared disorganized, and the overall lobular architecture was disrupted. Central veins exhibited dilation and congestion, while sinusoids showed marked dilatation and irregularity. Kupffer cells were increased in number and appeared activated, often clustering around inflamed areas. Hepatocytes displayed clear signs of cellular injury, including cytoplasmic vacuolation, nuclear condensation, and fragmented nuclei consistent with apoptosis and necrosis. Focal hepatocellular necrosis was observed, particularly in periportal and centrilobular regions. Early fibrotic changes were detected around portal areas, accompanied in some cases by bile duct proliferation and bile stasis, suggesting a reactive response to sustained injury. Additionally, portal and periportal regions exhibited prominent inflammatory cell infiltration, consisting mainly of lymphocytes and macrophages, with occasional neutrophils. As shown in Fig. 10c, quantitative histological analysis revealed significant differences in the number of inflammatory and pyknotic cells among the experimental groups. As compared to the control group, mice treated with Co-Cr alloy released ions exhibited a significant increase in inflammatory cells, accompanied by a significant rise in pyknotic hepatocytes. These alterations reflect extensive hepatocellular damage and marked structural disruption of liver tissue. These findings align with Amer et al. [87], who demonstrated that Co oxide nanoparticles caused vascular congestion, hepatocellular necrosis, and inflammatory infiltration in portal regions.

#### Effects of Co-Cr Alloy on Kidney Histopathology

**Fig. 11:**
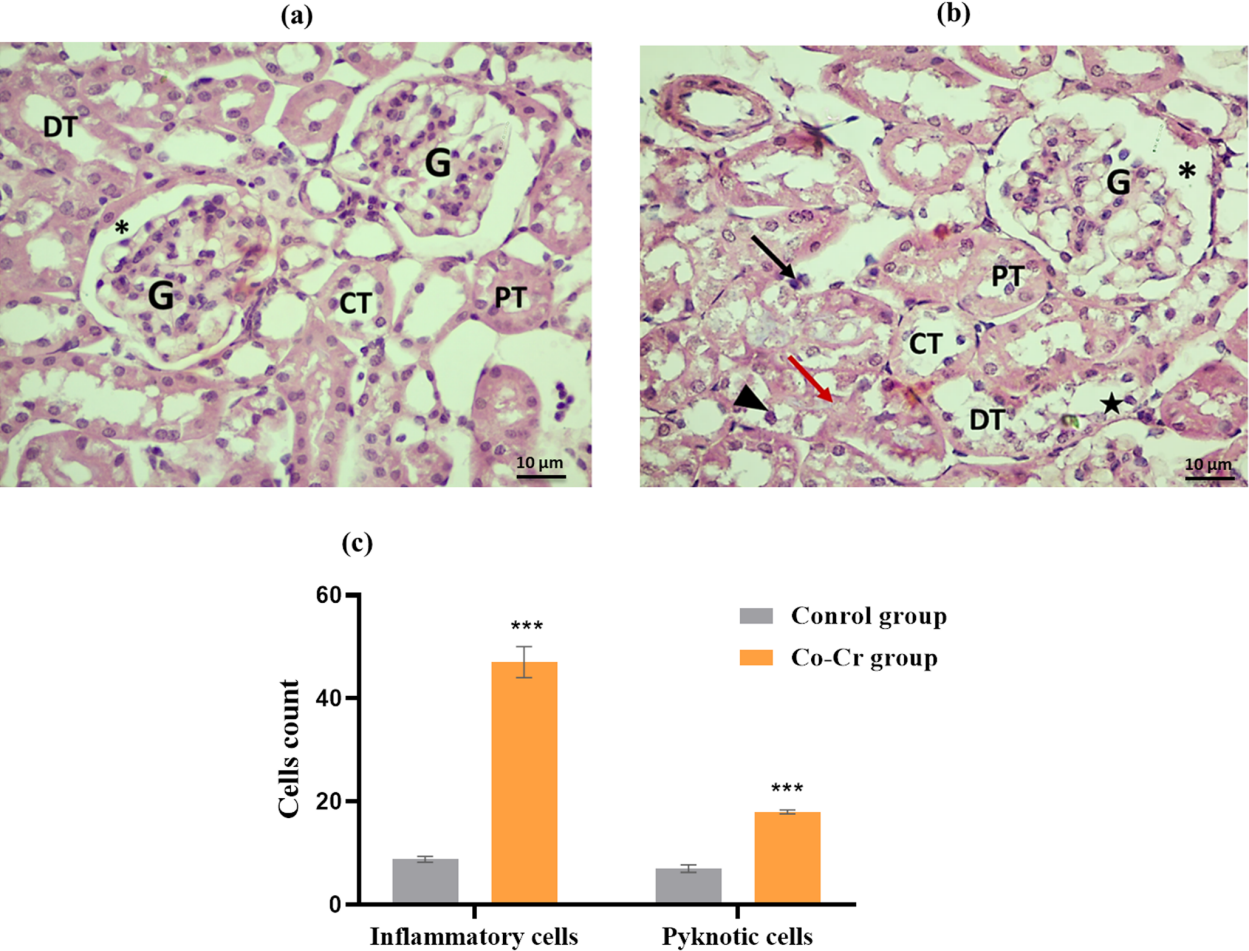
Photomicrographs of kidney from (**a**) Control group, (**b**) Co-Cr alloy ion released treated group stained with H&E, and (**c**) Quantification of inflammatory and pyknotic cells in kidney tissue across experimental groups. Bars represent the mean ± SE (*n* = 10). ***: significant vs. control at *p* < 0.001, using t-test. Control group shows normal renal corpuscles with normal glomeruli (G) and normal Bowman’s space (*); the distal tubules (DT) appeared having wider lumina lined by cuboidal cells with less acidophilic cytoplasm and rounded nuclei. Proximal (PT), and collected (CT) tubules are clearly distinguishable. Co-Cr alloy treated group demonstrates shrinkage of capillaries in the glomerulus (G) with the capsular space (*), degeneration in the epithelial cells (star) of both proximal (PT) and distal tubules (DT) in the form of dilation and cytoplasmic vacuolation with proteinous casts (red arrow) in lumen and pyknotic nuclei (head arrow); Slightly lymphocytic infiltration (black arrow) around the tubular structures (Scale bar 10 μm, magnification ×400)

Kidney tissues from the control group revealed preserved renal architecture with no detectable abnormalities (Fig. 11a). The renal cortex displayed a normal distribution of renal corpuscles, proximal and distal convoluted tubules, while the medullary region contained intact collecting ducts and loops of Henle. Both cortical and medullary structures showed intact epithelial linings, uniform cytoplasmic features, and well-maintained vascular organization, confirming the absence of inflammatory or degenerative alterations and reflecting a physiologically healthy renal state.

In contrast, mice treated with metal ions released from Co-Cr alloy exhibited clear histopathological alterations indicative of nephrotoxicity (Fig. 11b). Vascular dilation and congestion, often with intraluminal erythrocytes, were frequently observed, suggesting impaired renal hemodynamics. The interstitium showed mild infiltration of mononuclear inflammatory cells, particularly lymphocytes and macrophages, which were concentrated around tubules and blood vessels. This infiltration reflects an active immune response associated with Co-induced tissue injury. Glomeruli appeared distorted, with widened Bowman’s spaces, and numerous renal tubules showed pathological alterations, including epithelial desquamation, cytoplasmic vacuolization, tubular dilation, and interstitial edema. Furthermore, the quantitative histological data revealed that exposure to Co–Cr released ions led to a significant 5-fold increase in inflammatory cell infiltration, as well as a significant 2.5-fold rise in the number of pyknotic cells when compared with the control group (Fig. 11c). These changes collectively indicate tubular degeneration and loss of structural integrity. Comparable renal changes have been described in cobalt chloride–exposed rats, including mild tubular degeneration, areas of nephritis, and prominent inflammatory infiltration [71].

The persistent challenges of corrosion, ion release, and associated cytotoxic, inflammatory and systemic toxicity in traditional Co-Cr alloys highlight the need for advanced material strategies. Alloy modification, such as Boron-enriched Co-Cr, has been shown to reduce cytotoxicity and inflammatory response while supporting osteogenic differentiation and cell function critical for osseointegration [88]. In parallel, novel manufacturing techniques, including additive manufacturing of Co-Cr dental alloys improves their microstructural homogeneity and biocompatibility [89]. Surface modification strategies, such as anodization, plasma spray coatings, and nanotechnology-based layers can enhance biocompatibility by forming stable oxide barriers, and limiting ion leaching [90, 91]. Additionally, development of bioinert and biodegradable alloys, including refined titanium-niobium or titanium-molybdenum alloys and magnesium-based materials, offers safer alternatives, particularly for high-risk patients [17]. Collectively, these approaches highlight the importance of optimizing alloy composition, surface properties, and manufacturing techniques to maintain the mechanical benefits of metallic implants while minimizing long-term toxicity.

## Conclusion

The present study highlights the significant toxicological effects of ions released from Co–Cr dental alloys. At the cellular level, Co and Cr ions induced marked cytotoxicity in fibroblast cells, demonstrated by reduced cell viability and elevated expression of the pro-apoptotic marker BAX and the inflammatory mediators TNF-α and IL-6. Systemically, exposure to released ions resulted in oxidative stress, reflected by increased TBARS and DNA strand breakage in hepatic and renal tissues. Additionally, this oxidative burden was accompanied by a significant suppression of key antioxidant defense mechanisms, including CAT, GR, GST, and GSH. Serum biochemical analysis further revealed significant hepatic and renal dysfunction, with elevated ALT, AST, ALP, urea, and uric acid levels. Dyslipidemia, characterized by increased total cholesterol and triglycerides, also indicated systemic metabolic disturbance. Histopathological assessment corroborated these findings, showing inflammatory infiltration, hepatocellular degeneration, vascular congestion, and renal tubular alterations. Collectively, these results demonstrate that ions released from Co–Cr alloys exert substantial cytotoxic, oxidative, inflammatory, and genotoxic effects across both cellular and systemic levels. The observed biological alterations highlight the potential long-term health risks associated with ions released from Co-Cr dental alloys and underscore the importance of ongoing patient monitoring, especially for those with long-term restorations or pre-existing organ impairments. Additionally, these findings emphasize the need for developing corrosion-resistant and safer alternative materials for dental applications.

## Supplementary Information

Below is the link to the electronic supplementary material.

Supplementary Material 1

## Data Availability

All data generated or analyzed during this study are included in this published article.
