# Supplementary material for: Toxicological Assessment of Cobalt-Chromium Dental Alloys: Ion Release, Cytotoxicity, and Possible Systemic Effects
Source: Biol Trace Elem Res. 2026 Jan 8;204(6):3878–95. doi: 10.1007/s12011-025-04938-x (PMC13157445; doi:10.1007/s12011-025-04938-x)
Supplement: Supplementary file 1 — Supplementary Material 1 [file 12011_2025_4938_MOESM1_ESM.docx]

**Toxicological Assessment of Cobalt-Chromium Dental Alloys: Ion Release, Cytotoxicity, and Possible Systemic Effects**

**Ala AM Yahya ^a^, Gihan Hosny ^a^, Sabah G. El-Banna ^a^, Sara A. Alsakhawy ^a^**

^a^ *Department of Environmental Studies, Institute of Graduate Studies and Research, Alexandria University, Alexandria 21526, Egypt*

Corresponding author:

Dr. Sara A. Alsakhawy

E-mail address: [igsr.sarahatef@alexu.edu.eg](mailto:igsr.sarahatef@alexu.edu.eg)

| sample | **Bax (ng/mg protein)** |
| --- | --- |
| Control | 2.11 |
|  | 1.87 |
|  | 1.92 |
| Co | 3.87 |
|  | 3.49 |
|  | 4.12 |

| sample | **CAT (mU/mg protein)** |
| --- | --- |
| Control | 1.25 |
|  | 1.13 |
|  | 1.76 |
| Co | 5.24 |
|  | 4.18 |
|  | 3.27 |

| Sample | **SOD (mU/mg protein)** |
| --- | --- |
| Control | 2.23 |
|  | 2.17 |
|  | 1.82 |
| Co | 5.37 |
|  | 4.52 |
|  | 4.13 |

| sample | **IL-6 (pg/ml)** |
| --- | --- |
| Control | 102 |
|  | 89 |
|  | 97 |
| Co | 542 |
|  | 483 |
|  | 503 |

| sample | **TNF-α (pg/ml)** |
| --- | --- |
| Control | 33 |
|  | 42 |
|  | 36 |
| Co | 74 |
|  | 68 |
|  | 77 |
